# Synaptic connections formed by patchy projections of pyramidal cells in the superficial layers of cat visual cortex

**DOI:** 10.1007/s00429-017-1384-4

**Published:** 2017-02-27

**Authors:** German Koestinger, Kevan A. C. Martin, Stephan Roth, Elisha S. Rusch

**Affiliations:** Institute of Neuroinformatics, UZH/ETH, Winterthurerstrasse 190, 8057 Zurich, Switzerland

**Keywords:** Visual cortex, Pyramidal neuron, Orientation map, Dendrite, Synapse, Postsynaptic target

## Abstract

The present study is the first to describe quantitatively the patterns of synaptic connections made by the patchy network of pyramidal cell axons in the superficial layers of cat V1 in relation to the orientation map. Intrinsic signal imaging of the orientation map was combined with 3D morphological reconstructions of physiologically-characterized neurons at light and electron microscope levels. A Similarity Index (SI) expressed the similarity of the orientation domain of a given bouton cluster to that of its parent dendritic tree. Six pyramidal cells whose axons had a wide range of SIs were examined. Boutons were sampled from five local and five distal clusters, and from the linear segments that link the clusters. The synaptic targets were reconstructed by serial section electron microscopy. Of the 233 synapses examined, 182 synapses were formed with spiny neurons, the remainder with smooth neurons. The proportion of smooth neurons that were synaptic targets varied greatly (from 0 to 50%) between the cluster samples, but was not correlated with the SI. The postsynaptic density sizes were similar for synapses in local and distal clusters, regardless of their SI. This heterogeneity in the synaptic targets of single cells within the superficial layers is a network feature well-suited for context-dependent processing.

## Introduction

For Hubel and Wiesel (Hubel and Wiesel 1962), the radial cortical column is the ‘functional unit of cortex’ that most economically achieves the highly specific wiring needed to generate simple and complex cells in different layers. Linking these ‘columns’ are lateral connections that form a patchy network (Rockland and Lund 1982), which appears to be ubiquitous across all cortical areas in non-rodent species, including cat, tree shrew, monkey, and human (Douglas and Martin 2004). A common view is that the lateral connections link patches of common orientation preference, thus expressing a ‘like-to-like’ connection rule, achieved by means of a ‘fire-together-wire together’ mechanism (Gilbert and Wiesel 1989; Malach et al. 1994; Bosking et al. 1997; Sincich and Blasdel 2001). Given this consensus, we were surprised to discover that individual pyramidal cells in the superficial layers of the cat’s visual cortex form lateral clusters in a variety of orientation domains, including domains that were orthogonal to the parent cell’s preferred orientation (Martin et al. 2014a).

Various interpretations have been given to the functional role of these lateral connections. Electrophysiological mapping of visual receptive fields indicated the existence of ‘cross-orientation inhibition’, ‘surround inhibition’, and ‘end inhibition’ (Bishop et al. 1973; Hubel and Wiesel 1965; Morrone et al. 1982). For functions like end inhibition, the lateral connections would have to be between domains of the same orientation preference, whereas for cross-orientation inhibition, they would be between domains of orthogonal orientation preference, and this might explain the different patterns of lateral connections we uncovered (Martin et al. 2014a). All of these receptive field properties require that pyramidal cells lying some distance apart in the retinotopic representation mutually inhibit each other by means of intercalated inhibitory cells. Thus, the prediction of these models is that the predominant targets of the lateral projections are smooth GABAergic inhibitory neurons.

In their study of two physiologically-characterized pyramidal cells of the superficial layer of cat V1, Kisvarday et al. (1986) reported that over 90% of their synaptic targets in both local and distal clusters were other pyramidal cells. Thus, only small fractions were smooth GABAergic neurons, as characterized by ultrastructural characteristics and by post-embedding immunostaining for GABA. This pattern is consistent with reports of pyramidal cell synapses in cortical areas of the cat (LeVay 1988), and monkey (McGuire et al. 1991; Anderson et al. 2011). In mouse V1, however, smooth (GABAergic) neurons do form a far larger fraction of the targets of the local axon collaterals of superficial layer pyramidal cells than would be expected from the fraction of smooth neurons in the cortex (Bock et al. 2011; Bopp et al. 2014; Cossell et al. 2015). This apparent species difference remains to be explained.

A clustered lateral neural network, called a ‘daisy’ because of its *en face* appearance, is ubiquitous in the superficial layers of the cortex of higher mammals (Levitt et al. 1993; LeVay 1988; Douglas and Martin 2004). Only in the columnar visual cortex, however, is the functional architecture sufficiently well-defined to allow the structure of individual pyramidal cell axons to be correlated with functional maps like orientation preference (Karube and Kisvarday 2011; Martin et al. 2014a). The next step of examining the synaptic targets of these pyramidal cells in relation to the functional architecture has not yet been made, and this is the goal of the present study.

Here, we combined functional imaging, single cell electrophysiology, and intracellular labeling with 3D reconstructions at light and electron microscope level to discover the synaptic patterns of the daisy network. We discovered an unexpected high variability in the proportion of smooth neurons that were targets in a given cluster; this proportion was not related to the similarity in orientation domains of the cluster and the parent dendritic tree. Synapses were similar in size regardless of whether they were in a local or distal cluster or whether a basal or apical dendrite was the target. Only half of the boutons along the linear segments that linked the clusters actually formed synapses. Some linear segments were myelinated, but we estimated that this myelination contributes minimally to the conduction velocity. These findings shed new light on the longstanding puzzle of the role of the daisy in cortical computation.

## Results

The data were collected from six HRP-filled neurons obtained from five cats in which both optical recording and single unit electrophysiology had been performed before the neuron was filled intracellularly with HRP and reconstructed in 3D. To assist in correlating the physiology and anatomy, the cortex was sectioned parallel to the same plane as the optical imaging, which was near horizontal. To test whether the proportion of smooth neurons as targets was related to differences in orientation domain of the soma vs. its distal axonal clusters, we selected neurons whose distal clusters spanned the range from iso- to orthogonal orientation with respect to the parent neuron. A Similarity Index (SI) (Martin et al. 2014a) was used to give a quantitative estimate of the similarity in the orientation domain covered by the dendritic tree compared to the domain covered by given cluster. An SI of 1 indicated identical orientation tuning, and an SI of 0 indicated the boutons lay in an orientation domain that was tuned 90° to the domains of the dendrites. The local clusters typically arborised around and near the dendrites, so they have high SIs, whereas the SIs of distal clusters are far more variable (Martin et al. 2014a). We sampled extensive segments of axon from five local clusters, five distal clusters, and four linear segments of the axons that linked the local to the distal clusters and used correlated light microscopy (LM) and serial-section election microscopy (EM) to identify the target structures of 233 synapses. To reduce potential ambiguities in interpretation, we selected SIs that represented the extremes of the index—i.e., clusters that were located in orientation domains that were very similar to the dendritic tree (SI > 0.65) or those located in very different domains (SI < 0.35) and not those with intermediate indices (0.35 < SI < 0.65). We then used EM to determine whether the targets were spiny or smooth cells.

**Fig. 1:**
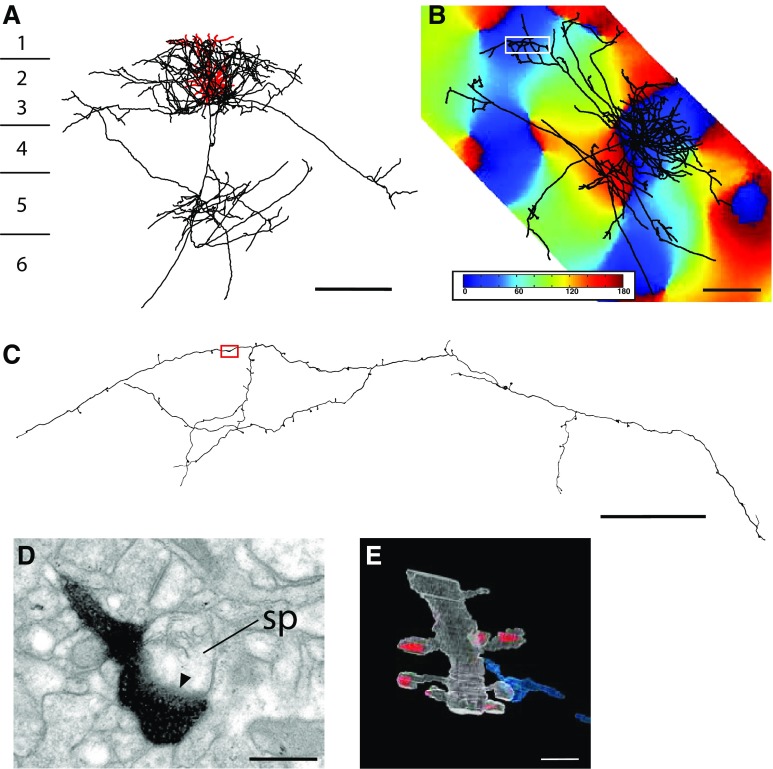
3D reconstruction of pyramidal cell with optical imaging map and sample EM image. **a** Coronal view of the dendrite (*red*) and axon (*black*), cortical laminae indicated to the *left* of the cell. **b** *Top view* of axon aligned with its corresponding optical imaging map. The *white box* marks the region of interest within the distal cluster examined in serial section EM. The SI of this cluster was 0.68, indicating that the dendrites and the distal axon cluster were located in similar domains of the orientation map. **c** LM reconstruction of the axon segment indicated in the *white box* in **b**. *Red box* indicates position of bouton section shown in **d. d** Electron micrograph of an electron-dense labeled bouton forming an asymmetric synapse (*solid arrowhead*) with an unlabeled spine (sp). **e** Partial reconstruction of dendrite and synapse shown in **d**. *Scale bars* **a, b** 500 µm; **c**–**e** 1 µm. The *colour code* for this and Fig. 2 indicates the orientation preference determined with moving gratings (see “Methods”): 0°/180° is horizontal, 90° is vertical

Figure 1 shows a reconstruction of a layer 3 pyramidal cell in coronal (Fig. 1a) and top view (Fig. 1b), with the orientation map (Fig. 1b) and a detail of the LM (Fig. 1c) and EM (Fig. 1d) of the distal axon. This neuron had that a dendritic tree was located in a horizontal domain of the orientation map. We sampled boutons from the local cluster (SI = 0.79), and from a distal cluster, whose SI of 0.68 indicated that it lay in domains that had the same orientation preference as the parent dendritic tree and the local cluster.

**Fig. 2:**
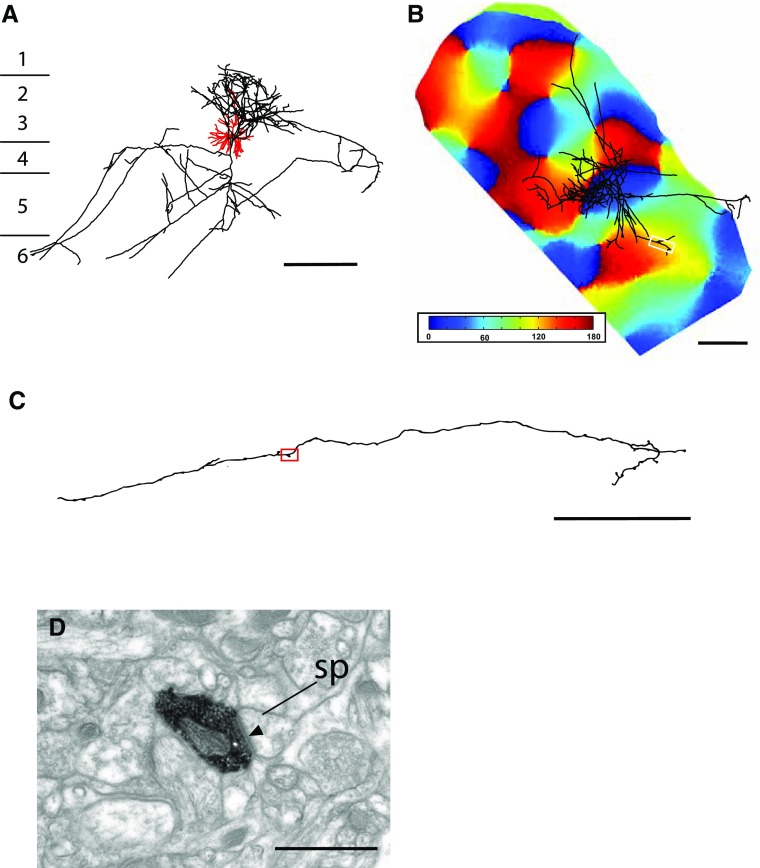
3D reconstruction of pyramidal cell with optical imaging map and sample EM image. **a** Coronal view of the dendrite (*red*) and axon (*black*) of pyramidal cell, cortical laminae are shown to the *left* of the cell. **b** *Top view* of the axon aligned with its corresponding optical imaging map. The *white box* marks the region of interest within the distal cluster examined in serial section EM. The SI of this cluster was 0.14, indicating that the dendrites and the distal axon cluster were located in dissimilar domains of the orientation map. **c** LM reconstruction of the axon segment indicated in the *white box* in **b**. *Red box* indicates position of bouton section shown in **d. d** Electron micrograph of an electron-dense labeled bouton forming an asymmetric synapse (*solid arrowhead*) with an unlabeled spine (sp). *Scale bars* **a, b** 500 µm; **c, d** 1 µm

All the synaptic data are derived from serial section EM reconstructions of segments of axon identified in the LM. An example of the correlated LM and EM of an en passant bouton formed by the distal cluster is illustrated in Fig. 1c and d. The electron micrograph indicates that synapse was an asymmetric Gray’s type 1 synapse (arrowhead indicates postsynaptic density), which is typical of pyramidal cell axons, and it was located on the head of dendritic spine containing spine apparatus. Reconstruction of a length of this postsynaptic dendrite revealed multiple spines (Fig. 1e), indicating that the target was another pyramidal cell, since these are the only source of spiny dendrites in this layer.

A very different pattern of lateral innervation is illustrated in Fig. 2 in that unlike the neuron in Fig. 1, the distal cluster we sampled was located in a domain, whose orientation preference was orthogonal to the parent cell’s local cluster and dendritic tree (Fig. 1b). The *bouton terminal* illustrated in the LM reconstruction shown in Fig. 1c formed an asymmetric, Gray’s type 1 synapse (arrowhead indicates postsynaptic density) with a dendritic spine (Fig. 1d), indicating that its target was a pyramidal cell. Spines formed the majority of targets of this distal cluster.

### Target dendrites

**Fig. 3:**
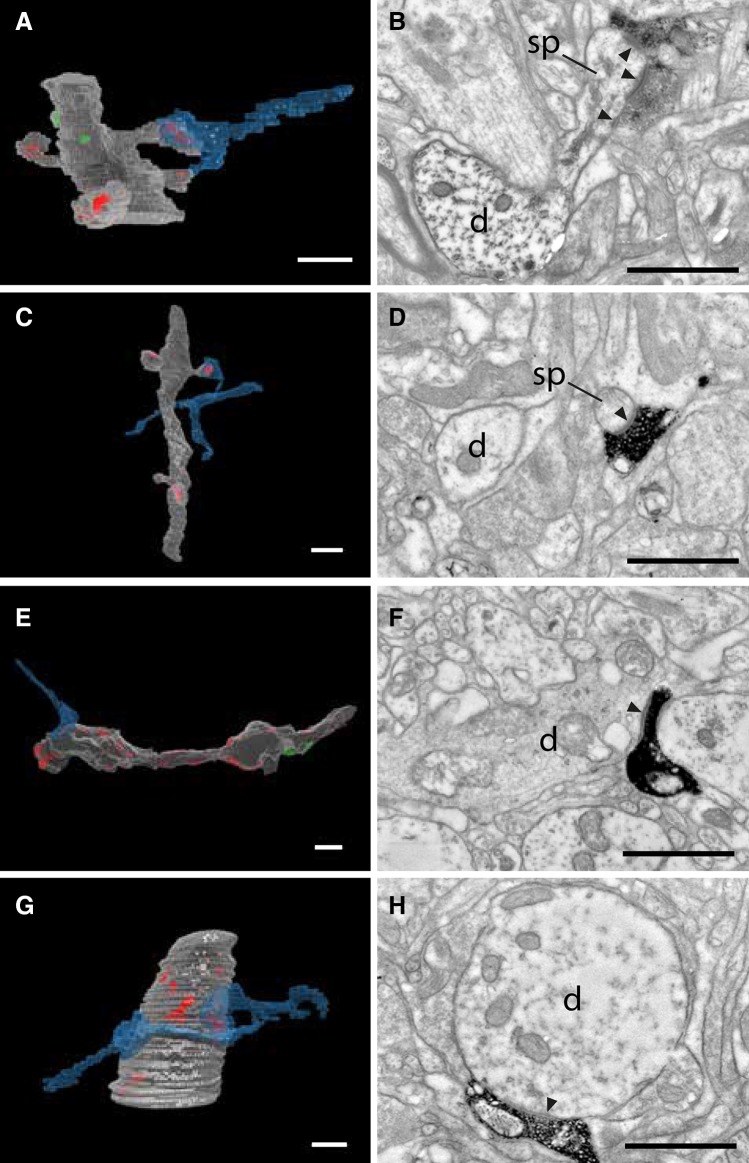
Range of dendritic targets of labeled axons. Pairs of images show rendered reconstruction from serial sections (**a, c, e, g**) and an example single section through labeled synapses (**b, d, f, h**). **a, b** Thick spiny dendrite, probably an apical dendrite, forms two synapses with the labeled axon (*arrowheads*). **c, d** Thin spiny dendrite forms an asymmetric synapse (*arrowhead*) with a labeled axon. **e, f** Smooth dendrite with distinctive beads typical of GABAergic neurons. **g, h** Large diameter smooth dendrite forming many shaft synapses with unlabeled boutons and with synapses formed by two boutons terminaux from the labeled axon. Labeled axons indicated in *blue*, asymmetric postsynaptic densities in *red*, symmetric synaptic densities in *green* for **a, c, e, g**. *d* dendrite, *sp* spine. *Scale bars* 0.5 µm

Virtually, all the target structures were dendrites. Only 5 of the 233 labeled synapses were formed with somata. The major targets were dendritic spines (174 synapses), followed by dendritic shafts (54 synapses). Most of the dendritic shafts (48) had the characteristics of smooth neurons in that they lacked spines and the shaft was covered with asymmetric synapses; 6 synapses formed with shafts of dendrites that bore spines. The majority of boutons along the axons were en passant, but *boutons terminaux* were also found. Examples of the variety of morphologies encountered are illustrated in Fig. 3. The axon in Fig. 3a and b formed two synapses with the spines of a thick dendrite, which, from its size and orientation, was probably the apical dendrite of a pyramidal cell. For comparison, a much thinner spiny dendrite is shown in Fig. 3c and d. A *bouton terminal* from the labeled axons formed a synapse with a small spine (arrowed in Fig. 3d). A beaded dendrite lacking spines and forming many asymmetric synapses (Fig. 3e, f) has the characteristics of smooth neurons. It formed one synapse with an en passant bouton. The dendrite (Fig. 3g, h) is also from a smooth neuron, and from its thickness, this segment is probably close to the soma of origin. It formed two synapses with two *boutons terminaux* originating from the labeled axon.

**Fig. 4:**
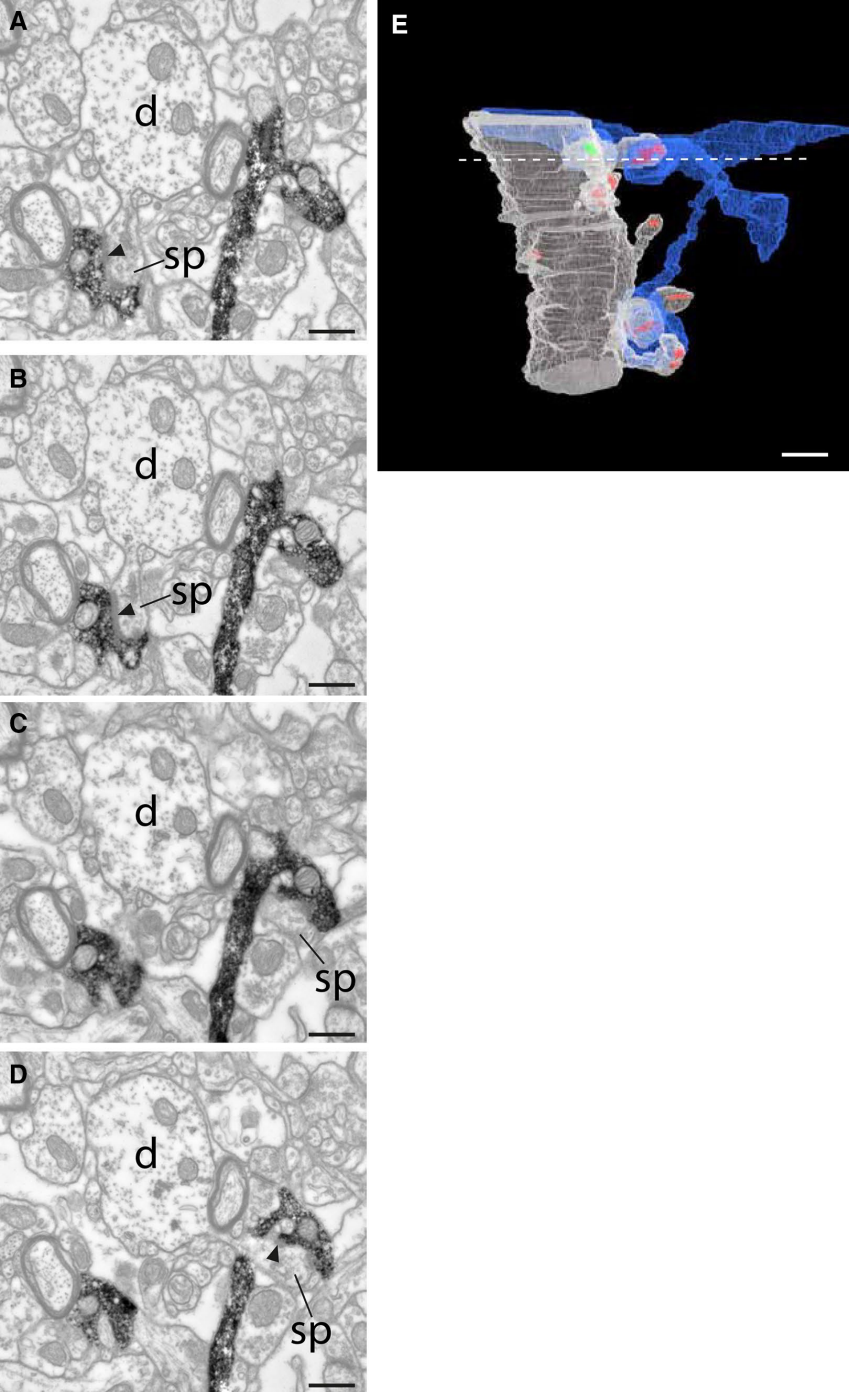
Axon forming a cluster of four synapses with an apical dendrite. **a**–**d** Electron micrograph series of a labeled axon (*black* stain in **a**–**d**) with boutons forming asymmetric synapses (*filled arrowheads*) with spines (sp) originating from the same dendrite (d). **e** 3D rendering of the dendritic segment (*light gray*) and the labeled axon (*blue*). The dendrite forms asymmetric synapses (*red*) and a symmetric synapse (*green*) with its presynaptic boutons. The labeled axon made four asymmetric synapses with dendritic spines (PSD areas 0.18, 0.16. 0.15, and 0.14 µm^2^). The *white dashed line* marks the sampled location of the electron micrographs **a**–**d**. *Scale bars* 0.5 µm

In most instances, an axon formed only one en passant synapse on the reconstructed segment of a target spiny dendrite, which is consistent with a geometry in which the straight trajectory of the axon makes an oblique angle with the postsynaptic dendrite. Multiple synapses are rare: in all we found four doublets, one triplet and one quadruplet (see examples in Figs. 3a and g, 4). In Fig. 4, the HRP-filled unmyelinated axon in a distal cluster formed a number of boutons in a small volume. Serial section reconstruction revealed that the axons formed four synapses with four different spines that were attached to the same parent dendrite. The serial sections illustrated (Fig. 4a–d) were taken through a bouton at the plane of the dotted line in the 3D reconstruction (Fig. 4e). The targets of two separate boutons seen in this plane of section were two unlabeled dendritic spines, which connected to a large diameter dendritic shaft by thin spine necks. The same axon formed asymmetric synapses with two other spines from the same dendrite, making four synapses in all.

Apical and basal dendrites were distinguished by their size (see Fig. 3a, b vs. Fig. 3c, d) and orientation. Because we sectioned in the horizontal plane, apical dendrites ran approximately orthogonal to the plane, whereas basal dendrites ran mostly obliquely within the sections. Thus, from its large diameter, radial trajectory, and spine bearing surface, the target of this unusual multi-synaptic cluster was most likely the apical dendrite of a pyramidal cell. In the cases illustrated in Figs. 3b and 4a–d, the fortuitous plane of sectioning enabled spine head, neck, and parent dendritic shaft to be visible in a single section. This view, however, was extremely rare. More often, the spine neck was sectioned obliquely, so that only a fragment appeared in each section, and because of the thinness of the spine neck, not all spine heads could be traced back reliably to their parent dendrites. The proportion of spines that could be traced back to the parent shaft varied from 20 to 85%, where the recovery correlated approximately with the quality of the tissue.

### Position of synapses on the dendritic tree

**Fig. 5:**
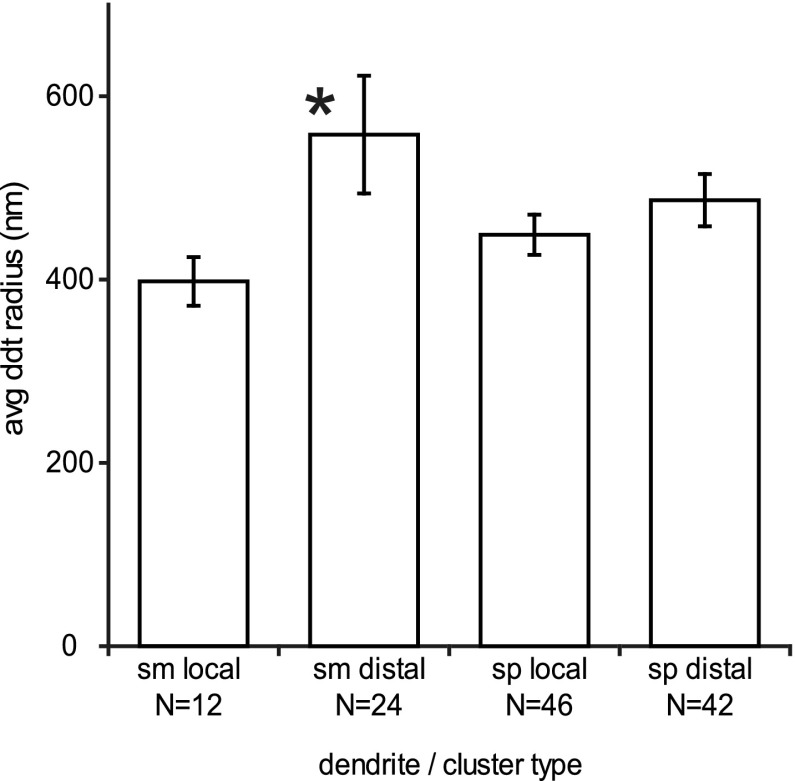
Histogram summarising average radii of the dendrites targeted by the local and distal clusters of the HRP-filled neurons. The *asterisk* indicates that the radii between smooth (sm) dendrites targeted by local and distal clusters are significantly different (two-tailed, two-sample *t* test *p* < 0.05). This was not the case for spiny (sp) dendritic targets. *Bars* indicate standard error. Number of dendrites sampled indicated beneath the histogram

We were eager to discover whether there was any evidence that local and distal portions of the axon formed their synapses with different parts of the dendritic tree of the postsynaptic neurons. One indication of the position on the dendritic tree is the radius of the target dendrites. As noted above, large radii would likely indicate an apical rather than a basal dendrite of a spiny cell or a more proximal location on the dendritic tree. Figure 5 shows that the radii of spiny dendrite targets in local vs. distal clusters were in fact similar (Fig. 5, right histogram). A significant difference was found for the smooth (putative GABAergic) dendrites (Fig. 5, left histogram), where the boutons in distal clusters formed their synapses with thicker smooth dendrites on average (e.g., Fig. 3g, h), indicating that these synapses are likely made closer to the somata of the smooth neurons that those in the local clusters.

### Synapse sizes and spine volumes

**Fig. 6:**
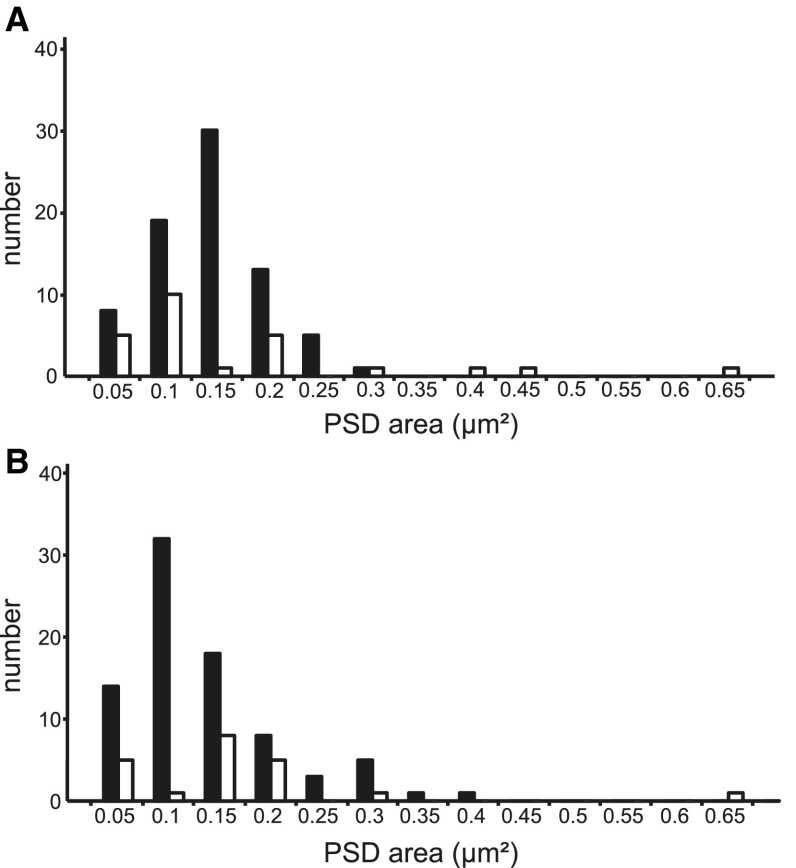
Distribution of areas (µm^2^) of the postsynaptic densities formed by labeled boutons. **a** Local clusters (*n* = 103 synapses). **b** Distal clusters (*n* = 101 synapses). *Black bars* indicate synapses formed with spiny neurons, and *white bars* indicate synapses formed with smooth neurons

The size of each synapse was assessed by measuring the area of the postsynaptic density (PSD) reconstructed from serial sections. The histograms in Fig. 6 show the PSD areas for synapses formed with smooth and spiny dendrites in local (Fig. 6a) and distal (Fig. 6b) clusters. The PSD sizes of synapses in the local and distal clusters were not statistically different. The distribution of the sizes of the PSDs formed with spines (dark bars) was also similar to those formed with dendritic shafts (open bars), although there were a few very large PSDs formed with shafts of smooth neurons.

**Fig. 7:**
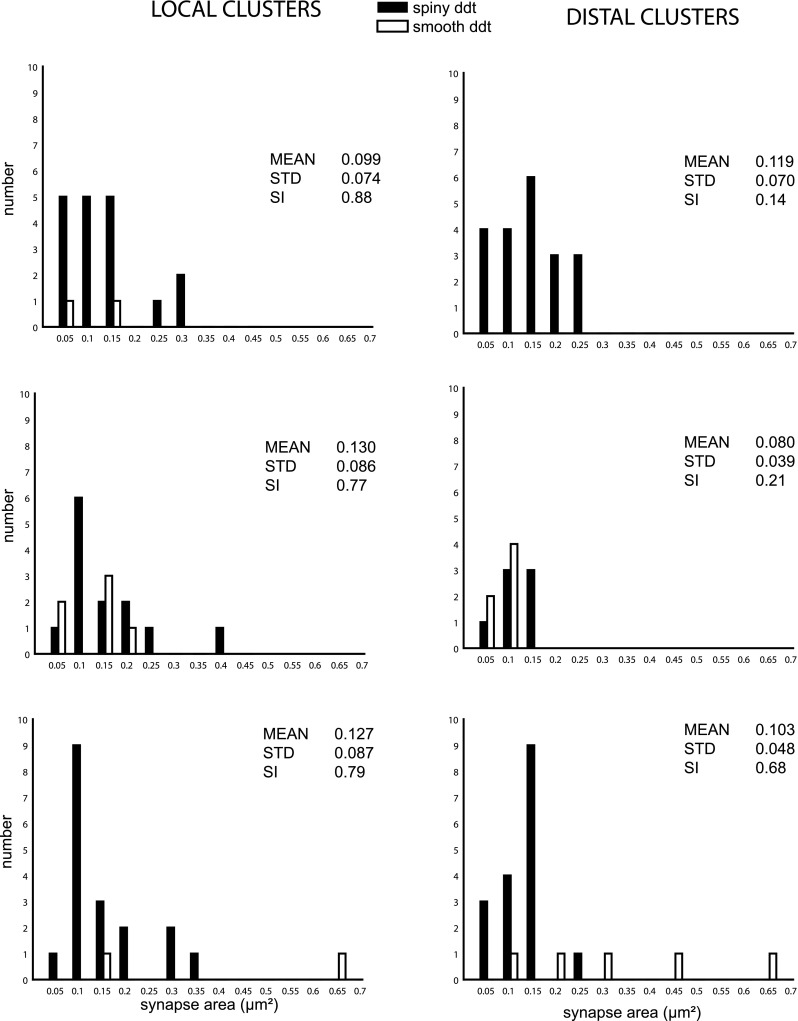
Relation of PSD size to SI for local and distal clusters. Labeled axons formed synapses with spiny dendrites (*black bars*) and smooth dendrites (*open bars*). Mean, standard deviation, and SI are indicated for the synapses sampled from three local (*left*) and three distal clusters (*right*)

Local clusters, which form in the vicinity of the dendritic tree, tend to have higher SIs (range 0.61–0.96) than distal clusters (range 0.13–0.96) (Martin et al. 2014a). Here, we examined at ultrastructural level whether the SI correlated with the size of the PSD formed with spiny and smooth dendrites. The results are given in Fig. 7 for the six clusters of known SI. The distributions of synapse areas for the spiny targets overlapped considerably and none were significantly different despite the large differences in the SI (SI range 0.14–0.88). The largest PSDs were found for smooth targets (see Fig. 6), but there was no significant correlation with the SI.

**Fig. 8:**
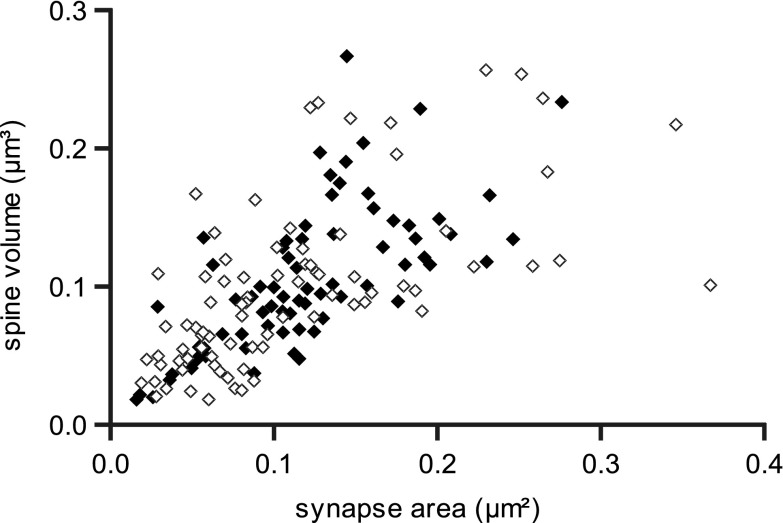
Relationship between PSD area (µm²) and the volume (µm³) of the associated spines. *Open diamonds* show values for PSD and spines from local clusters (Pearson Correlation, *r* = 0.63), and *filled diamonds* are PSD and spines from distal clusters (*r* = 0.68)

The size of the PSD is known to correlate with the size of the postsynaptic spine, but whether the slope of this relationship is the same for local and distal synapses is not known. Figure 8 gives a scatter plot of the data points for the local and distal spine synapses overlap completely and have similar correlation coefficients (Pearson’s correlation coefficient for local synapses *r* = 0.63; distal synapses *r* = 0.68).

### Proportion of spiny and smooth dendrite targets

**Fig. 9:**
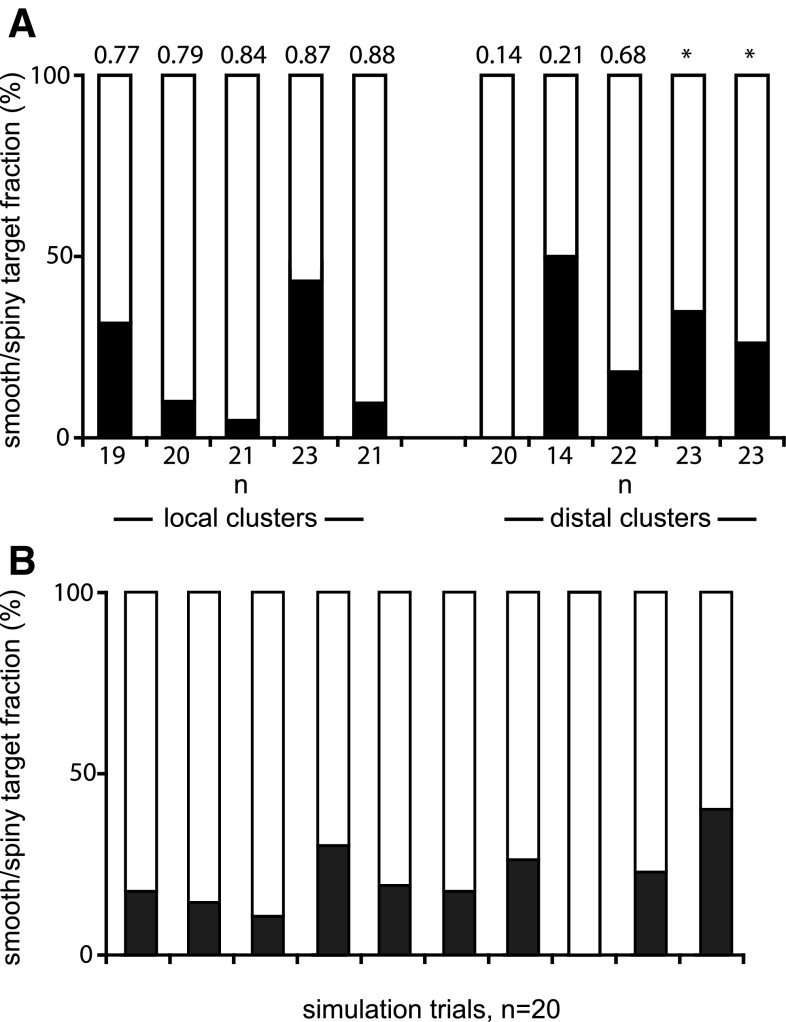
Proportion of spiny and smooth dendrite targets for experimental data (**a**) and simulated data (**b**). *Black bars* indicate synapses formed with smooth dendrites, and *white bars* indicate spiny dendrite targets. *Bars* in **a** are sorted in ascending SI (number on *top* of *bars*). *Asterisks* indicate two distal clusters that lay at the edge of the optical map, which made their similarity indices less reliable

The serial section reconstructions of segments of the postsynaptic dendrites allowed us to determine the proportion of synapses that formed with the two major classes of neurons—spiny and smooth neurons. The boutons were sampled from five local and five distal clusters. The SIs were also known for all of the local clusters and three distal clusters. These data are presented in the histograms of Fig. 9a. While it was expected that spiny dendrites would be the main postsynaptic targets, the high variance between clusters was unexpected. The wide range was spanned by the samples from both local and the distal clusters. In distal clusters, the range of spiny neurons as targets was 50–100%. For the local clusters, the range was similar: 52–95% spiny neurons as targets.

We then investigated whether the SI was related to the proportion of smooth and spiny dendrites formed within a particular cluster (Fig. 9a). The SIs of the local clusters indicated that they all lay in orientation domains that were similar to those of the parent dendritic tree (SI 0.77–0.88). This is expected, given that the local clusters form near and around the dendritic tree. The proportion of smooth dendrites that were targets, however, covered a very wide range (5–45% of targets). There was no relation of the SI to the proportion of smooth and spiny targets. This is most apparent in the two most extreme samples from the distal clusters, whose SIs (0.21 and 0.14) indicated that both clusters lay in orientation domains that were orthogonal to the orientation domains occupied by the parent dendritic tree. One of these distal clusters formed synapses only with spiny dendrites, while the other formed 50% of its synapses with spiny dendrites and 50% with smooth dendrites. In the other three samples of distal clusters, the proportion of spiny dendrites as targets lay between these extremes, giving percentages of 65, 72, and 82% (Fig. 9a).

To obtain an estimate of expected variance in target types, we simulated the distribution of spiny and smooth dendritic targets that would form possible synaptic targets of an axon that made a quasi-random walk through a 500 µm cube of neuropil, whose average composition and density of synaptic sites were determined from large disector counts (see “Methods”). The simulations (Fig. 9b) showed that the variance in spiny vs. smooth targets encountered in the quasi-random walk was of the same magnitude as the variance in the experimental data (Fig. 9a). Thus, we cannot exclude the possibility that synapse formation is stochastic at micron resolution.

### Analysis of neuropil around labeled boutons

**Fig. 10:**
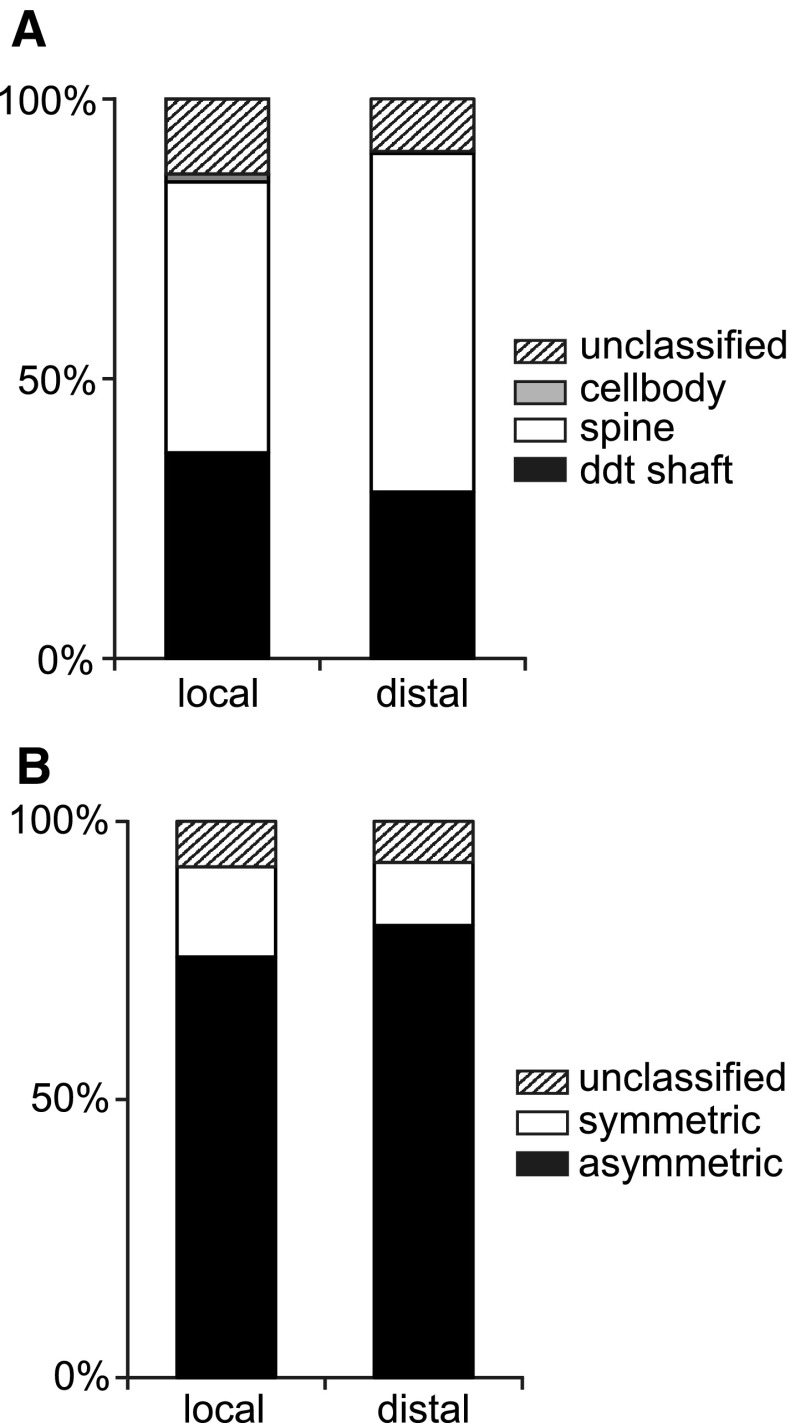
Quantification of neuropil targets (cell bodies, dendritic shaft, and spine) and synapses within local and distal clusters determined from unbiased disectors. **a** Proportions of targets of unlabeled synapses formed in the vicinity of a labeled synapse. Samples were taken from local and distal clusters. **b** Proportions of asymmetric and symmetric synapses formed in the vicinity of a labeled bouton forming a synapse

Given the wide variance in the proportion of smooth and spiny neurons that were synaptic targets of the labeled boutons, we analysed the neuropil immediately surrounding the labeled bouton. We determined the type of synapses (asymmetric or symmetric) and their targets for 205 synapses formed by unlabeled boutons in the vicinity of a labeled bouton in both local and distal clusters. The results of the unbiased disector counts are illustrated in the histograms of Fig. 10. As might be expected, most synapses were formed with spines (Fig. 10a) and were asymmetric (Fig. 10b). There was no difference in the average composition of the neuropil sampled around labeled boutons in local vs. distal clusters.

**Fig. 11:**
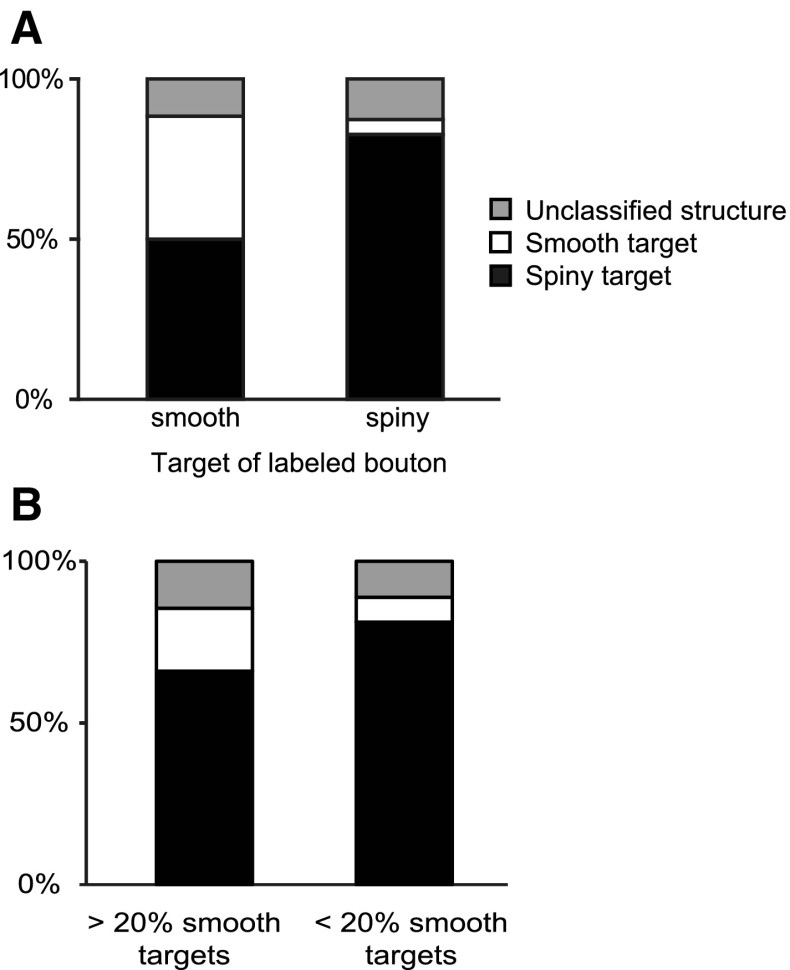
Relation of neuropil composition on distribution of targets. **a** Histograms of target types of synapses formed by unlabeled boutons in the neuropil. *Left bar* shows the identity of nearby target structures that formed synapses with unlabeled boutons in the region, where a labeled bouton formed a synapse with a smooth neuron. *Right bar* shows the composition of the targets of neuropil synapses when the labeled bouton formed a synapse with a spiny neuron. **b** Composition of target types in the neuropil for clusters which made more than 20% of their synapses with smooth neurons (*left bar*), and less than 20% of their synapses with smooth neurons (*right bar*)

The question we then asked is whether the composition of targets in the local neuropil was related to the type of synaptic target (spine, smooth dendritic shaft) of the labeled bouton? We made disector counts of the neuropil in the close vicinity of 60 labeled synapses (see “Methods”). Our analyses (Fig. 11) revealed that when the target of a given labeled bouton was a spiny dendrite, then a larger fraction of unlabeled boutons also formed synapses with spiny dendrites in the surrounding neuropil (Fig. 11a, right). Conversely, if the target was a smooth dendrite, then nearby unlabeled boutons also formed more synapses with smooth dendrites (Fig. 11a, left). These trends may to some extent reflect a bias generated by the availability of other synaptic targets on the same target dendrite, i.e., if the labeled axon formed, only one synapse with a smooth dendrite, then all the other unlabeled synapses formed with the same smooth dendrite will effectively bias the statistics of the local neuropil. The bias evens out considerably when we averaged across the samples that formed less than 20% of their synapses with smooth neuron and compared them with samples that formed more than 20% of their synapses with smooth neurons (Fig. 11b). These observations do raise an important question of how heterogenous or homogenous the neuropil is on the scales we are considering here, but our simulation indicated that considerable variance in the proportion of smooth and spiny neuron targets is statistically possible even with a homogenous average neuropil.

**Fig. 12:**
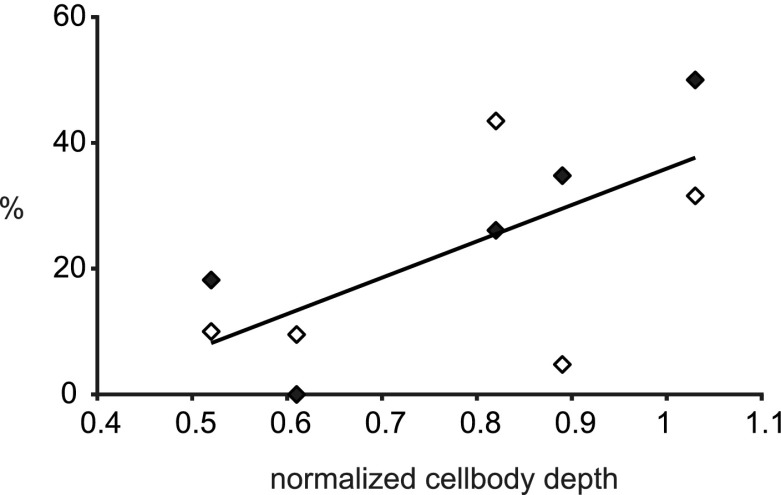
Relation of depth (*abscissa*) of parent pyramidal cell body to the proportion of smooth neurons (*ordinate*) with which it formed synapses. *Open diamonds* indicate data from local clusters, *filled diamonds* indicate data from distal clusters Between 14 and 23 synapses were used to calculate the proportion for each point. *Line* indicates Pearson correlation, *r* = 0.67

There was one further intriguing correlation that we did discover, which was that the proportion of smooth neurons that were targets of synapses formed by the ten clusters was related to the depth of the parent pyramidal cell within the superficial layers. From the 3D LM reconstructions, we observed that the deeper the cell body, the deeper the clusters it formed in the superficial layers. Figure 12 plots the cell body depth of each of the five neurons, each with samples from one local and one distal cluster. Depth in the graph is normalized: 0 is surface and 1 is the border between layers 3 and 4. Figure 12 shows that the deeper the cell body the larger the fraction of the synapses formed with smooth neurons (Pearson’s correlation coefficient *r* = 0.67).

### Linear axonal segments linking to distal clusters

The distal clusters typically arose from a branch of the main descending axon that traversed through the layers before entering the white matter. The laterally directed branches had a straight trajectory and formed only a few bouton-like swellings before branching to form the distal clusters that were, by contrast, densely studded with en passant boutons. This segment of axon connecting the main axon to the distal cluster we refer to as the ‘linear’ axonal segment (Binzegger et al. 2007). In the clusters, almost all boutons identified at LM level formed synapses, whereas when we examined the linear segments of the axon that connected the main axon to the distal bouton clusters, we found that of 50 swellings examined, only 28 actually formed synapses. Admittedly, many of the swellings were very small, and therefore, different in appearance at LM level from the larger boutons found in the clusters, which consistently formed synapses. The swellings that formed no synapses contained only mitochondria, no vesicles. The targets of boutons that did form synapses were spines (*n* = 22; PSD area mean 0.14, SD 0.09 µm^2^) and smooth dendrites (*n* = 6; PSD area mean 0.11, SD 0.07 µm^2^). The areas of the PSDs were not significantly different to those in the clusters.

### Myelin

While it is known that almost all axons in the white matter below V1 in adult cats are myelinated (Anderson et al. 1992), little is currently known of the degree of myelination of axons that form the lateral pathways within a cortical area. It was clear from our partial reconstructions of the clusters that the thin axons (mean diameter 0.18 µm) in the terminal arborisations were all unmyelinated. In four cells, we also examined in serial sections one of the linear segments that connected the main axon to the distal clusters. Two of the four linear segments were myelinated, two were not. The total thickness (axon plus myelin, uncorrected for a fixation shrinkage of 11%) of the myelinated segments varied in diameter from 0.15 to 0.56 µm (mean 0.30 µm, *n* = 34 measurements) and the myelin sheath varied in thickness from 0.040 to 0.080 µm (mean 0.048 µm).

Rushton (1951) defined the axon diameter divided by the total fiber diameter as the ‘*g*-ratio’ for myelinated axons. In the peripheral nervous system, he calculated that a *g*-ratio of 0.6 was optimal for maximizing the conduction velocity for a given diameter of fiber. This agreed well with the experimental measurements by Hursh (1939) of conduction velocity and myelin thickness in peripheral nerves of the cat. Extending Rushton’s theory by adding the constraint of volume to those of the biophysical parameters, Chomiak and Hu (2009) calculated an optimal *g*-ratio for CNS axons as 0.77, The average *g*-ratio of our axons of 0.75 lay close to this optimal value.

The question we now can answer is whether the degree of myelination we observed significantly increases the conduction velocity? The empirical diameter–velocity relationship for myelinated axons derived by Waxman and Bennett (1972) has a slope of 5.5 m/s per µm diameter (although they allow that the smallest diameter myelinated fibers would be better fit with a flatter slope). On their relationship, the maximum conduction velocity for our myelinated fibers of 0.35 µm diameter would be 1.9 m/s and about 15% slower for the unmyelinated fibers of the same diameter.

## Discussion

The lateral clusters of boutons formed by superficial layer pyramidal cells are a ubiquitous if enigmatic feature of the neocortex of higher mammals (Douglas and Martin 2004; Muir et al. 2011). With rare exceptions (Buzas et al. 2006), however, previous studies have analysed the ‘average’ projection pattern resulting from bulk labeling of many neurons. Very few studies involved complete LM reconstructions of physiologically identified single neurons, and even fewer have combined this with intrinsic signal imaging (Martin et al. 2014b). The present study is the first to combine intrinsic signal mapping with 3D morphological analyses at LM and EM level of single physiologically-characterized cells. Our results indicated that the axons of superficial layer pyramidal cells form synapses with variable proportions of smooth and spiny cell dendrites within the local cluster and the distal clusters. Our simulations indicated that even in the relatively homogenous neuropil we modeled, a high degree of variance in target type occurs if the axon makes its connections stochastically along a quasi-linear trajectory. The experimental variation in the proportion of smooth and spiny neuron targets was of the same magnitude as the simulations.

### Influence of orientation preference on target preference

Orientation selectivity is the most strongly expressed emergent property in the visual cortex of higher mammals, such as the cat. We discovered that at the resolution of single cells, the distal clusters of boutons do not follow a simple ‘like-to-like’ rule, where domains of the same orientation preference are connected. Instead, they formed distal clusters in a far more heterogeneous range of orientation domains (Martin et al. 2014a). We found that the proportion of target types was not related in any consistent way to the domain of the orientation map in which the cluster formed. Moreover, while the position of a cluster is likely determined according to some computational necessity (Martin et al. 2014a), the synapses within a given cluster may simply form opportunistically with any available site as the collaterals grow in their relatively straight trajectory through the neuropil (Braitenberg and Schüz 1991), as our simulations suggested.

Studies in vivo in the mouse have suggested that the synaptic connections between neurons with like spatial receptive fields are stronger than between neurons with unlike spatial receptive fields, as reflected in the amplitude of the excitatory postsynaptic potential subsequently measured in brain slices in vitro (Cossell et al. 2015) and in the larger area of the PSD (Lee et al. 2016). In contrast, we found no significant differences in PSD sizes between local and distal clusters, regardless of their SIs; the degree of similarity of orientation preference alone does not control the size of the PSD in the daisy network of cat V1.

### Innervation of smooth (GABAergic) neurons

The closest comparable study of synapses formed by pyramidal cell axons in the superficial layers of cat V1 is that of Kisvarday et al. (1986). In their study of two pyramidal cells located near the border of layers 3 and 4, they found that the proportion of spine targets in the superficial clusters varied between 82 and 93%. Some of the target dendritic shafts they tested were not GABA-immunoreactive and they concluded that no more than 5% of all the synapses of the two cells were formed with GABAergic smooth neurons. We found that clusters that were located in upper tier of layer 3 formed about 10% of their synapses with smooth dendrites on average. By contrast, deeper clusters (which also originated from parent somata lying nearer the layer 3/4 border) formed a higher proportion of their synapses (up to 50% in one cluster) with smooth neurons. This likely reflects some sublaminae difference in available smooth neurons. This within-laminar variation has not been seen in V1 of the mouse, where superficial layer pyramidal neurons target disproportionately smooth (GABAergic) neurons (up to 50% of targets; Bock et al. 2011; Bopp et al. 2014; Lee et al. 2016). One consequence of the lack of orientation columns in the mouse is GABAergic neurons in the mouse are weakly orientation tuned (Zariwala et al. 2011), reflecting their convergent excitatory input, whereas in the cat, the GABAergic neurons have the same orientation tuning as their neighbouring pyramidal cells (Keller and Martin 2015).

### Lateral conduction velocity

Although ‘striate cortex’ (V1, area 17) takes its name from the myelin stria in the middle layers, the myelination of axons in the gray matter has received little attention. It is known that the main axon of superficial layer pyramidal cells myelinates in the gray matter en route to the white matter (Martin and Whitteridge 1984a), but it was not known before this study that some of the linear segments that connect the main axon to the distal clusters are also myelinated. Our question was then whether this degree of myelination would significantly increase the lateral conduction velocity?

In Rushton’s theory (Rushton 1951), the threshold for myelination was an axon diameter of 1 mm for below that he calculated that unmyelinated axons of the same diameter conduct faster. In the central nervous system (CNS), axons and the myelin sheaths are thinner than in the peripheral nerves and the *g*-ratio measured in a variety of CNS axons is fairly constant—in the range 0.64–0.84 (Bishop et al. 1971; Chomiak and Hu 2009; Waxman and Bennett 1972). The *g*-ratio of our myelinated axons (0.75) lay in this range. Calculations of the conduction velocity in cat visual cortex based on indirect physiological measurements have produced a range of 0.1–0.36 m/s [0.36 m/s (Hirsch and Gilbert 1991); 0.2 m/s (Grinvald et al. 1994); 0.1 m/s (Grinvald et al. 1994); and 0.2–0.5 m/s (Bringuier et al. 1999; Benucci et al. 2007)], which suggest that the horizontal transmission in cat V1 is by means of very thin unmyelinated axons.

Using the empirical diameter–velocity relationship derived by Waxman and Bennett (1972), we estimated that the maximum conduction velocity for our myelinated fibers of 0.35 µm diameter would be 1.9 m/s, and about 15% slower for unmyelinated fibers of the same diameter. This estimate of conduction velocity is an order of magnitude faster than the calculations based on indirect physiological measurements mentioned above. The discrepancy is like due to other factors such as synaptic delays and integration times, which make the conduction velocity based on indirect estimates that seem slower than they actual. The difference in transmission time between myelinated and unmyelinated axons over the 1 mm distance between soma and distal cluster is marginal. This myelination may have more to do with factors like increased security of transmission than actually increasing conduction velocity.

### Role of lateral clusters

The role of the lateral clusters in V1 of higher mammals has been discussed primarily in terms of context-dependent and Gestalt perceptual phenomena (for reviews, see Martin 1988; Gilbert and Wiesel 1992), but lateral clusters have been implicated in many other functions, all of which require very specific connections. For example, they are thought to provide co-linear or co-circular facilitation and receptive field modulation (Stettler et al. 2002; Chisum et al. 2003; Angelucci and Bullier 2003), texture and curve continuity (Field et al. 1993; Bosking et al. 1997; Schmidt et al. 1997; Ben-Shahar and Zucker 2004), illusory contours (Gilbert and Wiesel 1992), contour integration (Stettler et al. 2002), colour constancy (Gilbert and Wiesel 1992), feature binding, and scene segmentation through promotion of synchronized oscillatory activity (Gray and Singer 1989; Engel et al. 1990; Gray et al. 1990; Benucci et al. 2007), and representation of focal orientation discontinuities such as junctions or corners (Sillito et al. 1995). How can all these phenomena be assigned to the daisy network? One resolution is to suppose that the lateral connections do not ‘drive’ their targets, but only modulate the sensitivity of the neuron to another ‘driving’ input, without modifying its selectivity or receptive field properties (Hirsch and Gilbert 1991). We found no structural evidence to suggest that distal synapses are weaker that the local synapses, so we suppose their postsynaptic effects are equivalent.

A reasonable concern regarding our analysis might be the uncertainty of the location of the soma of the postsynaptic target neuron in respect to the orientation map. We minimized this concern by analyzing synapses belonging to individual clusters having functional similarities at the extreme ends of the Similarity Index: i.e., clusters that were located in orientation domains that were very similar to the dendritic tree, SI > 0.65, or those located in very different domains, SI < 0.35, and excluded those with intermediate indices or which lay near pinwheels. Since most synapses on a dendritic tree lie within 50–100 µm (in the lateral dimension) from the parent soma, it is not the case that the target neurons lay in a completely different orientation domain to the cluster. Thus, we can be reasonably confident that the ‘effective SI’ of the target pyramidal cells would not be very different from the SI of the cluster itself.

Various strategies have been used to examine what effects lateral connections might have on receptive field properties. The common strategy of reducing a response by adaptation through long exposure to one stimulus produces modest shifts in the peaks of the orientation tuning curves away from the adapting stimulus (a ‘repulsive’ shift; Dragoi et al. 2000, 2001; Muller et al. 1999; Felsen et al. 2002). Very small effects were also seen in spike-timing conditioning paradigms (Yao and Dan 2001; Yao et al. 2004). In the adaptation and conditioning experiments, the site of the alteration is, of course, unknown. The most direct examination of the action of the lateral projections was the experiment in which small clusters of neurons in the superficial layers were inactivated pharmacologically. Inactivation of neurons lying 250–500 µm from neurons recorded in superficial layers of cat visual cortex resulted in a shift or broadening of the orientation tuning, or a loss in direction selectivity (Crook et al. 1997, 1998; Girardin and Martin 2009). These data indicate that the lateral connections made by the superficial layer pyramidal cells do have a significant, spatially specific, influence on their targets, rather than simply being a means of broadcasting a modulating gain-control signal.

### Recurrent circuits

Superficial layer pyramidal cells in cat V1 connect massively with one another (Douglas et al. 1989; Douglas and Martin 1991). Binzegger et al. (Binzegger et al. 2004) estimated that the connections between superficial layer pyramidal cells alone constitute 20% of all the excitatory synapses in cat V1. Thus, it might be expected that their primary effect is to amplify a preferred response, so that the effect of inactivating a cluster of neurons by GABA iontophoresis would be to reduce the amplitude of the stimulus-evoked response in their targets. Such reductions are observed, but paradoxically, so are increases in firing rate (Girardin and Martin 2009). The decreases in firing rate are likely due to inactivation of excitatory inputs, whereas the increases presumably reflect disinhibition. This disinhibition is most likely due to removal of an excitatory drive to the smooth neurons that directly inhibit the target neurons. Our present data provide the structural basis for both outcomes. The important inference is that the orientation selective responses of a superficial layer pyramidal cell are not simply inherited from orientation selective layer four neurons, but is generated locally and dynamically by an ensemble of orientation specific connections (Martin et al. 2014a), which include both feedforward and recurrent components.

Physiological studies also reveal something of the complexity of the afferent input to V1 cells. In analyzing in vivo intracellular responses to visual stimuli in cat V1 neurons, Monier et al. (2003) and Fournier et al. (2014) discovered a rich diversity in the feature selectivity of the afferents that produced subthreshold responses. They found that 60% of their sample of cells had the same orientation tuning for excitation and inhibition, but for the remaining 40%, the inhibitory and excitatory components differed in their tuning preferences. In a fine-grained analysis of the filter properties of V1 receptive fields, Fournier et al. (2014) found that cells with simple- and complex-like receptive fields had additional non-linear (‘complex’) subunits provided by excitatory and inhibitory afferents. Of the excitatory complex subunits, 42% had orientation preferences that were oblique or orthogonal to the orientation selectivity of the simple receptive field. For the inhibitory non-linear subunits, 73% had orientation preferences that were oblique or orthogonal to that of the simple receptive field. While these subunits do not translate into the single afferent inputs we have studied, it is clear that the sources of input to single neurons arise from neurons that span a great variety of spatiotemporal receptive fields. The heterogeneity of synaptic targets seen in the present study and by our related structure–function study showing that the distal bouton clusters form in widely differing orientation domains (Martin et al. 2014a) provides a structural basis for the physiological heterogeneity observed.

The heterogeneous connection patterns we have described may explain why the signal and noise correlations between adjacent neurons in the same functional column are so small (Martin and Schroder 2013; Cohen and Kohn 2011) despite the clustering of common attributes, like orientation selectivity, in the cortices of higher mammals. Importantly, the existence of lateral clusters in many areas of cortex in higher mammals suggests that these mechanisms are not unique to visual processing, but are a design feature of cortical processing (Douglas and Martin 2004).

Large resources are now being devoted to studying the structure of cortical circuits by means of ‘dense’ reconstructions from massive EM data sets obtained from a single species—the mouse. Given the tiny dimensions over which such dense reconstructions are currently feasible, it is clear that dense reconstruction is not a viable technique for studying the local circuits of the dimensions we have reported here. Furthermore, the sample size for dense reconstructions is typically* n* = 1, so vital information about the statistical variance of the circuits is lacking. Thus, the approach we have pursued here of sparse reconstructions of the synaptic targets of a common pyramidal cell type from a number of experiments, provides valuable measures of this variance at cellular and synaptic resolution, and our LM and EM analyses point to computational reasons why such variance exists.

## Methods

The experiments were carried out under licenses granted to KACM by the Kantonales Veterinaeramt of Zurich. Full details of the methods for optical recording of the orientation maps and the single unit recordings and intracellular injections of horseradish peroxidase, together with cell reconstruction and analysis methods are given in Martin et al. (Martin et al. 2014a) and are briefly described here; only the additional methods for the electron microscopy and simulations are described in detail.

### Surgery

Five adult cats of either sex were maintained under general anaesthesia for the duration of the experiment. After craniotomy, the cats were given a continuous i.v perfusion of muscle relaxants (gallamine triethiodide, Sigma Aldrich, CH, 13 mg/kg/h, and (+)-tubocurarine chloride hydrate, Sigma, 1 mg/kg/h). General anaesthesia was maintained with (30%/70%). Halothane (0.5–1.5%) and continuous i.v. infusion of alphadalone/alphaxalone (Saffan, Glaxo) sufficient to maintain the electroencephalogram (EEG) in a light sleep (spindling) state. EEG, ECG, heart rate, arterial blood pressure, end-tidal CO_2_, and rectal temperature were monitored continuously during the entire experiment. A thermistor-controlled heating blanket maintained the cat’s rectal temperature at 37°. The eyes protected with gas permeable contact lenses and were refracted to focus on the tangent screen.

### Recording

Glass micropipettes were filled with a 4% solution of Horseradish Peroxidase (HRP, Roche) in 0.05 M Tris and 0.2 M KCl at pH 7.9 and then beveled to impedances between 40 and 88 MΩ (mean 72 MΩ ± 12). Extracellular receptive fields (RFs) were hand-plotted and classified (S or C, simple or complex; Martin and Whitteridge 1984b). In successful attempts to impale the neuron, its receptive field was checked to be sure that the extracellular receptive field belonged to the actual neuron and HRP was iontophoresis (Martin and Whitteridge 1984a).

### Optical imaging

For optical imaging, a metal chamber (Optical Imaging, Inc) was fixed with dental cement to the skull around the rim of the craniotomy. The camera (CS8310BC, Teli, Japan) was then focused on the surface brain through a macroscope (Ratzlaff and Grinvald 1991) using a wavelength of 546 nm (isobestic for hemoglobin). The camera angle was adjusted, so that a larger surface of cortex was in focus and the angle of the macroscope was noted with respect to the stereotaxic planes. A digital image of the cortical surface was taken to record the blood vessel pattern with normal light. Then, the illumination wavelength was changed to 700 nm and the camera focused down 450 µm below brain surface (with the 700 nm illumination this corresponded to 600 µm below surface). Image acquisition was synchronized with visual stimulation, and five data frames of 600 ms duration were acquired during stimulus presentation (each data frame was the sum of 15 camera frames). Data acquisition was done with an Imager 3001 VSD + setup (Optical Imaging, Inc) and using the software VDAQ (Optical Imaging, Inc).

Visual stimuli consisted of square wave gratings of eight different orientations (0°, 22.5°, 45°, 67.5°, 90°, 112.5°, 135°, and 157.5°) with 100% contrast, spatial frequency of 1 cycle/degree and temporal frequency of 1 degree/s. During inter-stimulus intervals, the next stimulus was presented as stationary. The visual stimuli were displayed to the cat in a random order. All the visual stimuli were programmed in Matlab (MATHworks) and presented using a VSG2/5 graphics card (Cambridge Research).

Single orientation maps (termed as ‘single maps’) were calculated in two ways. Responses from individual orientations (summed activity of 26–52 trials) were divided by the summed response to all stimuli (cocktail blank). Alternatively, differential maps were calculated in which responses from individual orientations were divided by the orthogonal orientation (Bonhoeffer and Grinvald 1996).

### Alignment of brain sections with the orientation map

Before the perfusion, up to six reference penetrations were made with empty glass pipettes in the stereotaxic coordinate frame. The position of the reference penetrations was noted on the blood vessel pattern and in the stereotaxic coordinates. At the end of the penetration, when the tip of the micropipette was at a depth of 2 mm below the brain surface, the shaft was cut across a few mm above the brain surface and remained in place for perfusion (Phillips et al. 1971). These tracks were identified in histological sections and were used to align the reconstructed neurons with the optical imaging maps.

### Fixation and histology

At the end of the experiment, the cat was euthanized and perfused transcardially with normal 0.9% NaCl solution, followed by a room-temperature solution of 4% paraformaldehyde, 0.3% glutaraldehyde, and 15% saturated solution of picric acid in 0.1 M PB pH 7.4. After vibratome sectioning at 80 µm, the HRP was revealed using 3-diaminobenzidine tetrahydrochloride (DAB) with nickel intensification. After assessment by light microscopy (LM), the sections containing labeled neurons were further processed for electron microscopic analyses. These sections were treated with 1% osmium tetroxide in 0.1 M PB, dehydrated through alcohols (1% uranyl acetate in the 70% alcohol) and propylene oxide, and flat mounted in Durcupan (Fluka) on glass slides.

### Reconstructions

Neurons were reconstructed in 3D using a microscope (100×, Olympus BX-51) combined with a motorized stage (MicroBrightField Inc., USA) and the aid of the Neurolucida software (Version 8.0, MicroBrightField Inc., USA). The reconstruction of one neuron took approximately 100 h. While reconstructing the axon, each bouton was tagged with a marker. The borders of cortical layers were determined in tangential sections on the basis of light microscopic characteristics visible in the osmium-treated tissue, such as relative neuron and fiber densities, neural soma size, HRP-filled dendrites, the presence of large pyramidal cells at the border region of layer 3 and 4, and giant pyramidal cells of Meynert in layer 5b.

### Correlated light and electron microscopy

Serial light micrographs were taken from the osmicated sections at different magnifications and the blood vessel pattern surrounding labelled neurons was reconstructed using TrakEM2 (Cardona et al. 2012). The dendritic arbour and the axon the neuron of interest was then reconstructed first in 2D using a drawing tube attached to a light microscope, and then in 3D from serial light micrographs using TrakEM2.

For electron microscopy, the tissue was serially re-sectioned at 60 nm thickness and collected on Pioloform coated single slot copper grids. The axon of labeled neurons was then found in the ultrathin sections and synapse connectivity between labeled axons and neuropil targets investigated with transmission electron microscopy (TEM). Synapses and associated structures were classified using conventional criteria.

### Counts of unlabeled synapse targets in the neuropil

We estimated of the percentage of dendritic targets (spines or shafts) using the physical disector method (Sterio 1984). The disector was composed of two serial sections of known thickness (50 nm) separated by one intervening section. Synapses that disappeared from reference to lookup section were counted and the target was classified as dendritic spine or shaft. Both sections were used as reference and lookup doubling the number of disectors per site. Electron micrographs were collected at a magnification of (13,500×, pixel size 2.5 nm) with a digital camera (11 mega pixels, Morada, Soft Imaging Systems). Four sets of counts were performed. The first set was done on randomly selected location in the neuropil surroundings the labeled neurons. The disectors had a size of 5 × 5 µm and were sampled from the first intact section of every fourth grid (each grid contained eight sections on average). The sampling sites (five sites per animal) and grids were selected according to a systematic random sampling scheme. The second set was done on the neuropil surrounding the labelled boutons of recorded neurons. The counts were made for randomly selected labeled boutons (60 sites sampled in total) from local and distal clusters. The disectors had a size of 7.3 × 4.9 µm. We examined all synapses in the EM micrograph that contained the first postsynaptic density of the labeled bouton, which disappeared in the second-next section and vice versa. With this procedure, we made sure that there is equal sampling for objects with different sizes (as in the disector method).

A third set of images were taken from a single animal to map the exact 2D locations of the synapses for use in the Monte Carlo simulations described below. Three randomly located large disectors were made. The disectors had a size of 12.7 µm × 6.8 µm, and the sampling sites (eight sampling sites) and sections were chosen according to a systematic random sampling scheme (da Costa et al. 2009).

### Monte Carlo simulations

A Monte Carlo analysis was made to test whether the observed statistics of synaptic targets by labeled axons could be due to a random process (Braitenberg and Schüz 1991). We made two assumptions: first that in a large enough neuropil, volume synapses are distributed uniformly, so that the sum of all synapses represents all possible targets; second that the growth of an axon in a cluster can be approximated by a biased random walk through the neuropil volume. The location of targets in the virtual neuropil followed a uniform distribution as found in the biological data obtained from five large disectors (30 µm × 30 µm). We ran a simulation in MATLAB (Mathworks) of an axon growing through a cube of this virtual neuropil of size 500 µm on a side. When an axon approach within 1 µm of a target spine or smooth dendritic shaft, it was scored as a synapse. Each simulation was run 10,000 times with the parameters from each labeled neuron/neuropil and it was terminated when the virtual axon reached the number of synapses reconstructed for each labeled neuron. The result of each simulation was the proportion of smooth dendritic targets on the virtual axon.

## References

[CR1] Anderson JC, Dehay C, Friedlander MJ, Martin KAC, Nelson JC (1992). Synaptic connections of physiologically identified geniculocortical axons in kitten cortical area 17. Proc R Soc B Biol Sci.

[CR2] Anderson JC, Kennedy H, Martin KA (2011). Pathways of attention: synaptic relationships of frontal eye field to V4, lateral intraparietal cortex, and area 46 in macaque monkey. J Neurosci.

[CR3] Angelucci A, Bullier J (2003). Reaching beyond the classical receptive field of V1 neurons: horizontal or feedback axons?. J Physiol Paris.

[CR4] Ben-Shahar O, Zucker S (2004). Geometrical computations explain projection patterns of long-range horizontal connections in visual cortex. Neural Comput.

[CR5] Benucci A, Frazor RA, Carandini M (2007). Standing waves and traveling waves distinguish two circuits in visual cortex. Neuron.

[CR6] Binzegger T, Douglas RJ, Martin KA (2004). A quantitative map of the circuit of cat primary visual cortex. J Neurosci.

[CR7] Binzegger T, Douglas RJ, Martin KA (2007). Stereotypical bouton clustering of individual neurons in cat primary visual cortex. J Neurosci.

[CR8] Bishop GH, Clare MH, Landau WM (1971). The relation of axon sheath thickness to fiber size in the central nervous system of vertebrates. Int J Neurosci.

[CR9] Bishop PO, Coombs JS, Henry GH (1973). Receptive fields of simple cells in the cat striate cortex. J Physiol.

[CR10] Bock DD, Lee WC, Kerlin AM, Andermann ML, Hood G, Wetzel AW, Yurgenson S, Soucy ER, Kim HS, Reid RC (2011). Network anatomy and in vivo physiology of visual cortical neurons. Nature.

[CR11] Bonhoeffer T, Grinvald A, Toga AW, Mazziotta JC (1996). Optical imaging based on intrinsic signals: the methodology. Brain mapping: the methods.

[CR12] Bopp R, Macarico da Costa N, Kampa BM, Martin KA, Roth MM (2014). Pyramidal cells make specific connections onto smooth (GABAergic) neurons in mouse visual cortex. PLoS Biol.

[CR13] Bosking WH, Zhang Y, Schofield B, Fitzpatrick D (1997). Orientation selectivity and the arrangement of horizontal connections in tree shrew striate cortex. J Neurosci.

[CR14] Braitenberg V, Schüz A (1991). Anatomy of the cortex: statistics and geometry. Studies of brain function.

[CR15] Bringuier V, Chavane F, Glaeser L, Fregnac Y (1999). Horizontal propagation of visual activity in the synaptic integration field of area 17 neurons. Science.

[CR16] Buzas P, Kovacs K, Ferecsko AS, Budd JM, Eysel UT, Kisvarday ZF (2006). Model-based analysis of excitatory lateral connections in the visual cortex. J Comp Neurol.

[CR17] Cardona A, Saalfeld S, Schindelin J, Arganda-Carreras I, Preibisch S, Longair M, Tomancak P, Hartenstein V, Douglas RJ (2012). TrakEM2 software for neural circuit reconstruction. PLoS One.

[CR18] Chisum HJ, Mooser F, Fitzpatrick D (2003). Emergent properties of layer 2/3 neurons reflect the collinear arrangement of horizontal connections in tree shrew visual cortex. J Neurosci.

[CR19] Chomiak T, Hu B (2009). What is the optimal value of the *g*-ratio for myelinated fibers in the rat CNS? A theoretical approach. PLoS One.

[CR20] Cohen MR, Kohn A (2011). Measuring and interpreting neuronal correlations. Nat Neurosci.

[CR21] Cossell L, Iacaruso MF, Muir DR, Houlton R, Sader EN, Ko H, Hofer SB, Mrsic-Flogel TD (2015). Functional organization of excitatory synaptic strength in primary visual cortex. Nature.

[CR22] Crook JM, Kisvarday ZF, Eysel UT (1997). GABA-induced inactivation of functionally characterized sites in cat striate cortex: effects on orientation tuning and direction selectivity. Vis Neurosci.

[CR23] Crook JM, Kisvarday ZF, Eysel UT (1998). Evidence for a contribution of lateral inhibition to orientation tuning and direction selectivity in cat visual cortex: reversible inactivation of functionally characterized sites combined with neuroanatomical tracing techniques. Eur J Neurosci.

[CR24] da Costa NM, Hepp K, Martin KA (2009). A systematic random sampling scheme optimized to detect the proportion of rare synapses in the neuropil. J Neurosci Methods.

[CR25] Douglas RJ, Martin KA (1991). A functional microcircuit for cat visual cortex. J Physiol.

[CR26] Douglas RJ, Martin KA (2004). Neuronal circuits of the neocortex. Annu Rev Neurosci.

[CR27] Douglas RJ, Martin KAC, Whitteridge D (1989). A canonical microcircuit for neocortex. Neural Comput.

[CR28] Dragoi V, Sharma J, Sur M (2000). Adaptation-induced plasticity of orientation tuning in adult visual cortex. Neuron.

[CR29] Dragoi V, Rivadulla C, Sur M (2001). Foci of orientation plasticity in visual cortex. Nature.

[CR30] Engel AK, Konig P, Gray CM, Singer W (1990). Stimulus-dependent neuronal oscillations in cat visual cortex: inter-columnar interaction as determined by cross-correlation analysis. Eur J Neurosci.

[CR31] Felsen G, Shen YS, Yao H, Spor G, Li C, Dan Y (2002). Dynamic modification of cortical orientation tuning mediated by recurrent connections. Neuron.

[CR32] Field DJ, Hayes A, Hess RF (1993). Contour integration by the human visual system: evidence for a local “association field”. Vis Res.

[CR33] Fournier J, Monier C, Levy M, Marre O, Sari K, Kisvarday ZF, Fregnac Y (2014). Hidden complexity of synaptic receptive fields in cat V1. J Neurosci.

[CR34] Gilbert CD, Wiesel TN (1989). Columnar specificity of intrinsic horizontal and corticocortical connections in cat visual cortex. J Neurosci.

[CR35] Gilbert CD, Wiesel TN (1992). Receptive field dynamics in adult primary visual cortex. Nature.

[CR36] Girardin CC, Martin KA (2009). Inactivation of lateral connections in cat area 17. Eur J Neurosci.

[CR37] Gray CM, Singer W (1989). Stimulus-specific neuronal oscillations in orientation columns of cat visual cortex. Proc Natl Acad Sci USA.

[CR38] Gray CM, Engel AK, Konig P, Singer W (1990). Stimulus-dependent neuronal oscillations in cat visual cortex: receptive field properties and feature dependence. Eur J Neurosci.

[CR39] Grinvald A, Lieke EE, Frostig RD, Hildesheim R (1994). Cortical point-spread function and long-range lateral interactions revealed by real-time optical imaging of macaque monkey primary visual cortex. J Neurosci.

[CR40] Hirsch JA, Gilbert CD (1991). Synaptic physiology of horizontal connections in the cat’s visual cortex. J Neurosci.

[CR41] Hubel DH, Wiesel TN (1962). Receptive fields, binocular interaction and functional architecture in the cat’s visual cortex. J Physiol.

[CR42] Hubel DH, Wiesel TN (1965). Receptive fields and functional architecture in two nonstriate visual areas (18 and 19) of the cat. J Neurophysiol.

[CR43] Hursh JB (1939). Conduction velocity and diameter of nerve fibers. Am J Physiol.

[CR44] Karube F, Kisvarday ZF (2011). Axon topography of layer IV spiny cells to orientation map in the cat primary visual cortex (area 18). Cereb Cortex.

[CR45] Keller AJ, Martin KA (2015). Local circuits for contrast normalization and adaptation investigated with two-photon imaging in cat primary visual cortex. J Neurosci.

[CR46] Kisvarday ZF, Martin KA, Freund TF, Magloczky Z, Whitteridge D, Somogyi P (1986). Synaptic targets of HRP-filled layer III pyramidal cells in the cat striate cortex. Exp Brain Res.

[CR47] Lee WC, Bonin V, Reed M, Graham BJ, Hood G, Glattfelder K, Reid RC (2016). Anatomy and function of an excitatory network in the visual cortex. Nature.

[CR48] LeVay S (1988). Patchy intrinsic projections in visual cortex, area 18, of the cat: morphological and immunocytochemical evidence for an excitatory function. J Comp Neurol.

[CR49] Levitt JB, Lewis DA, Yoshioka T, Lund JS (1993). Topography of pyramidal neuron intrinsic connections in macaque monkey prefrontal cortex (areas 9 and 46). J Comp Neurol.

[CR50] Malach R, Tootell RB, Malonek D (1994). Relationship between orientation domains, cytochrome oxidase stripes, and intrinsic horizontal connections in squirrel monkey area V2. Cereb Cortex.

[CR51] Martin KA (1988). The Wellcome Prize lecture. From single cells to simple circuits in the cerebral cortex. Q J Exp Physiol.

[CR52] Martin KA, Schroder S (2013). Functional heterogeneity in neighboring neurons of cat primary visual cortex in response to both artificial and natural stimuli. J Neurosci.

[CR53] Martin KA, Whitteridge D (1984). Form, function and intracortical projections of spiny neurones in the striate visual cortex of the cat. J Physiol.

[CR54] Martin KA, Whitteridge D (1984). The relationship of receptive field properties to the dendritic shape of neurones in the cat striate cortex. J Physiol.

[CR55] Martin KA, Roth S, Rusch ES (2014). Superficial layer pyramidal cells communicate heterogeneously between multiple functional domains of cat primary visual cortex. Nat Commun.

[CR56] Martin KAC, Roth S, Rusch ES (2014). Superficial layer pyramidal cells communicate heterogeneously between multiple functional domains of cat primary visual cortex. Nat Commun.

[CR57] McGuire BA, Gilbert CD, Rivlin PK, Wiesel TN (1991). Targets of horizontal connections in macaque primary visual cortex. J Comp Neurol.

[CR58] Monier C, Chavane F, Baudot P, Graham LJ, Fregnac Y (2003). Orientation and direction selectivity of synaptic inputs in visual cortical neurons: a diversity of combinations produces spike tuning. Neuron.

[CR59] Morrone MC, Burr DC, Maffei L (1982). Functional implications of cross-orientation inhibition of cortical visual cells. I. Neurophysiological evidence. Proc R Soc Lond B Biol Sci.

[CR60] Muir DR, Da Costa NM, Girardin CC, Naaman S, Omer DB, Ruesch E, Grinvald A, Douglas RJ (2011). Embedding of cortical representations by the superficial patch system. Cereb Cortex.

[CR61] Muller JR, Metha AB, Krauskopf J, Lennie P (1999). Rapid adaptation in visual cortex to the structure of images. Science.

[CR62] Phillips CG, Powell TP, Wiesendanger M (1971). Projection from low-threshold muscle afferents of hand and forearm to area 3a of baboon’s cortex. J Physiol.

[CR63] Ratzlaff EH, Grinvald A (1991). A tandem-lens epifluorescence macroscope: hundred-fold brightness advantage for wide-field imaging. J Neurosci Methods.

[CR64] Rockland KS, Lund JS (1982). Widespread periodic intrinsic connections in the tree shrew visual cortex. Science.

[CR65] Rushton WA (1951). A theory of the effects of fibre size in medullated nerve. J Physiol.

[CR66] Schmidt KE, Goebel R, Lowel S, Singer W (1997). The perceptual grouping criterion of colinearity is reflected by anisotropies of connections in the primary visual cortex. Eur J Neurosci.

[CR67] Sillito AM, Grieve KL, Jones HE, Cudeiro J, Davis J (1995). Visual cortical mechanisms detecting focal orientation discontinuities. Nature.

[CR68] Sincich LC, Blasdel GG (2001). Oriented axon projections in primary visual cortex of the monkey. J Neurosci.

[CR69] Sterio DC (1984). The unbiased estimation of number and sizes of arbitrary particles using the disector. J Microsc.

[CR70] Stettler DD, Das A, Bennett J, Gilbert CD (2002). Lateral connectivity and contextual interactions in macaque primary visual cortex. Neuron.

[CR71] Waxman SG, Bennett MV (1972). Relative conduction velocities of small myelinated and non-myelinated fibres in the central nervous system. Nat New Biol.

[CR72] Yao H, Dan Y (2001). Stimulus timing-dependent plasticity in cortical processing of orientation. Neuron.

[CR73] Yao H, Shen Y, Dan Y (2004). Intracortical mechanism of stimulus-timing-dependent plasticity in visual cortical orientation tuning. Proc Natl Acad Sci USA.

[CR74] Zariwala HA, Madisen L, Ahrens KF, Bernard A, Lein ES, Jones AR, Zeng H (2011). Visual tuning properties of genetically identified layer 2/3 neuronal types in the primary visual cortex of cre-transgenic mice. Front Syst Neurosci.

